# *MIQuant* – Semi-Automation of Infarct Size Assessment in Models of Cardiac Ischemic Injury

**DOI:** 10.1371/journal.pone.0025045

**Published:** 2011-09-30

**Authors:** Diana S. Nascimento, Mariana Valente, Tiago Esteves, Maria de Fátima de Pina, Joana G. Guedes, Ana Freire, Pedro Quelhas, Perpétua Pinto-do-Ó

**Affiliations:** 1 Instituto de Engenharia Biomédica (INEB), Universidade do Porto, Porto, Portugal; 2 Instituto de Ciências Biomédicas Abel Salazar, Universidade do Porto, Porto, Portugal; 3 Faculdade de Engenharia, Universidade do Porto, Porto, Portugal; 4 Departamento de Epidemiologia Clínica, Medicina Preditiva e Saúde Pública, Faculdade de Medicina da Universidade do Porto, Porto, Portugal; Brigham & Women's Hospital - Harvard Medical School, United States of America

## Abstract

**Background:**

The cardiac regenerative potential of newly developed therapies is traditionally evaluated in rodent models of surgically induced myocardial ischemia. A generally accepted key parameter for determining the success of the applied therapy is the infarct size. Although regarded as a gold standard method for infarct size estimation in heart ischemia, histological planimetry is time-consuming and highly variable amongst studies. The purpose of this work is to contribute towards the standardization and simplification of infarct size assessment by providing free access to a novel semi-automated software tool. The acronym *MIQuant* was attributed to this application.

**Methodology/Principal Findings:**

Mice were subject to permanent coronary artery ligation and the size of chronic infarcts was estimated by area and midline-length methods using manual planimetry and with *MIQuant*. Repeatability and reproducibility of *MIQuant* scores were verified. The validation showed high correlation (*r* ^midline length^ = 0.981; *r* ^area^ = 0.970 ) and agreement (Bland-Altman analysis), free from bias for midline length and negligible bias of 1.21% to 3.72% for area quantification. Further analysis demonstrated that *MIQuant* reduced by 4.5-fold the time spent on the analysis and, importantly, *MIQuant* effectiveness is independent of user proficiency. The results indicate that *MIQuant* can be regarded as a better alternative to manual measurement.

**Conclusions:**

We conclude that *MIQuant* is a reliable and an easy-to-use software for infarct size quantification. The widespread use of *MIQuant* will contribute towards the standardization of infarct size assessment across studies and, therefore, to the systematization of the evaluation of cardiac regenerative potential of emerging therapies.

## Introduction

Cardiovascular disease is a leading cause of morbidity and mortality worldwide. Heart failure due to ischemic coronary artery disease is currently the most common cardiac disorder and it correlates with a worse prognosis [1], [2]. The physiological, histological and molecular changes associated with clinical ischemic heart disease have been clarified with the use of experimental models of myocardial infarction (MI) developed in both large animals, including dogs and swine, as well as in small rodents [3], [4]. The latter are more applicable for high-throughput screening of novel therapeutic approaches, due to the easy maintenance, short reproductive cycle and to the latest advances in gene-targeting and transgenic technologies. In recent years, the evaluation of cardiac regenerative potential of newly developed therapies, as is the case of gene-delivery and transplantation of stem/progenitor-cells, has been primarily explored in rat and mouse models of surgically-induced myocardial ischemia [2], [5], [6], [7], [8]. The so-called left anterior descending (LAD) coronary artery ligation is the prominent model in these studies, and the infarct size has been considered a key parameter for assessing the success of the novel therapy. A strong correlation between the infarction size and the functional and hemodynamic alterations following myocardial infarction is generally observed [9], [10], [11] and therefore considered a fundamental measure in the assessment of the morphological and functional consequences of infarction.

In studies involving an experimental MI setting, the calculation of the infarct size is typically evaluated by histological measurements of either: (a) the endocardial and epicardial length [10], [12], (b) the midline length [10], (c) the endocardial length [9] or (d) the area [13] of infarcted *versus* non-infarcted left-ventricle (LV) regions. Despite the widespread use of the aforementioned approaches, the infarct size can vary depending on the used method [10], [14] and therefore no direct comparison can be withdrawn across laboratories. Moreover, several aspects of MI size quantification that can also account for infarct size variation are inconsistent across studies and not always clearly defined, e.g. the number of sections used for the calculation, the histological staining and criteria used to identify the infarcted region. Thus, the purpose of the present work is to contribute towards standardization and simplification of the infarct size assessment in experimental models of MI by making available, as freeware, an easy-to-use semi-automatic software application, which we developed and validated at the “bench”. This tool will contribute for the systematization of the evaluation of cardiac regenerative potential of newly developed therapies. The acronym *MIQuant* that stands for *MI quantification* was attributed to the herein software application.

## Methods

### Animals

Male and female C57BL/6 mice aged 8 to 12 weeks were used for this study. All the procedures were subjected to approval by the IBMC-INEB (Instituto de Biologia Molecular e Celular – Instituto de Engenharia Biomédica) Animal Ethics Committee and to the National Direção Geral de Veterinária (permit no: 022793), and are in conformity with the Directive 2010/63/EU of the European Parliament. Humane endpoints were followed in accordance to the Organisation for Economic Co-operation and Development (OECD) Guidance Document on the Recognition, Assessment, and Use of Clinical Signs as Humane Endpoints for Experimental Animals Used in Safety Evaluation (2000).

### Surgical Induction of Myocardial Infarction

MI was experimentally induced by ligation of the LAD coronary artery as described elsewhere [13] with minor alterations. Following anesthesia by intraperitoneal injection (ip) of medetomidine (Sededorm, 1 mg/Kg) and ketamine (Clorketam, 75 mg/kg), animals were subjected to endotracheal intubation and were mechanically ventilated using a small-animal respirator (Minivent 845, Harvard Apparattus). Animals were maintained on warming pads during surgical procedure and until full recovery to prevent hypothermia. Under a stereomicroscope (Leica EZ4, Leica Microsystems) the heart was exposed (Ø 5–7 mm) via left thoracotomy on the third intercostal space and the pericardial sac was gently disrupted. After identification of the LAD coronary artery a non-absorbable 7-0 suture (Silkam®, B. Braun) was passed under the artery and the ligation was performed. The intercostal incision was closed by an absorbable 6-0 suture (Safil®, B. Braun) and surgical staples were used for skin closure. Anesthesia was reverted by atipamezole (ip, Revertor, 5 mg/Kg) and analgesia was achieved by butorphanol (ip, Butador, 1 mg/Kg). Analgesia and fluid therapy were performed by ip delivery of butorphanol (Butador, 1 mg/kg) and 5% glucose physiological saline, respectively. This procedure was repeated every 12 h up to 72 h post-surgery or until full animal recovery.

For organ collection animals were deeply anesthetized by ip injection of pentobarbital (Eutasil, 70 mg/kg). At 21days post-surgery, hearts were harvested, briefly washed in phosphate buffer saline and fixed in 10% Formalin neutral buffer (VWR BDH & Prolabo) up to 24 hours prior to paraffin-embedding. The sampling procedure herein described results on hearts arrested at variable stages of heart cycle, which may contribute to increased variability of infarct size. Whenever normalization is a requirement, hearts should be arrested in diastole following injection with potassium chloride.

### Histological procedures

**Figure 1:**
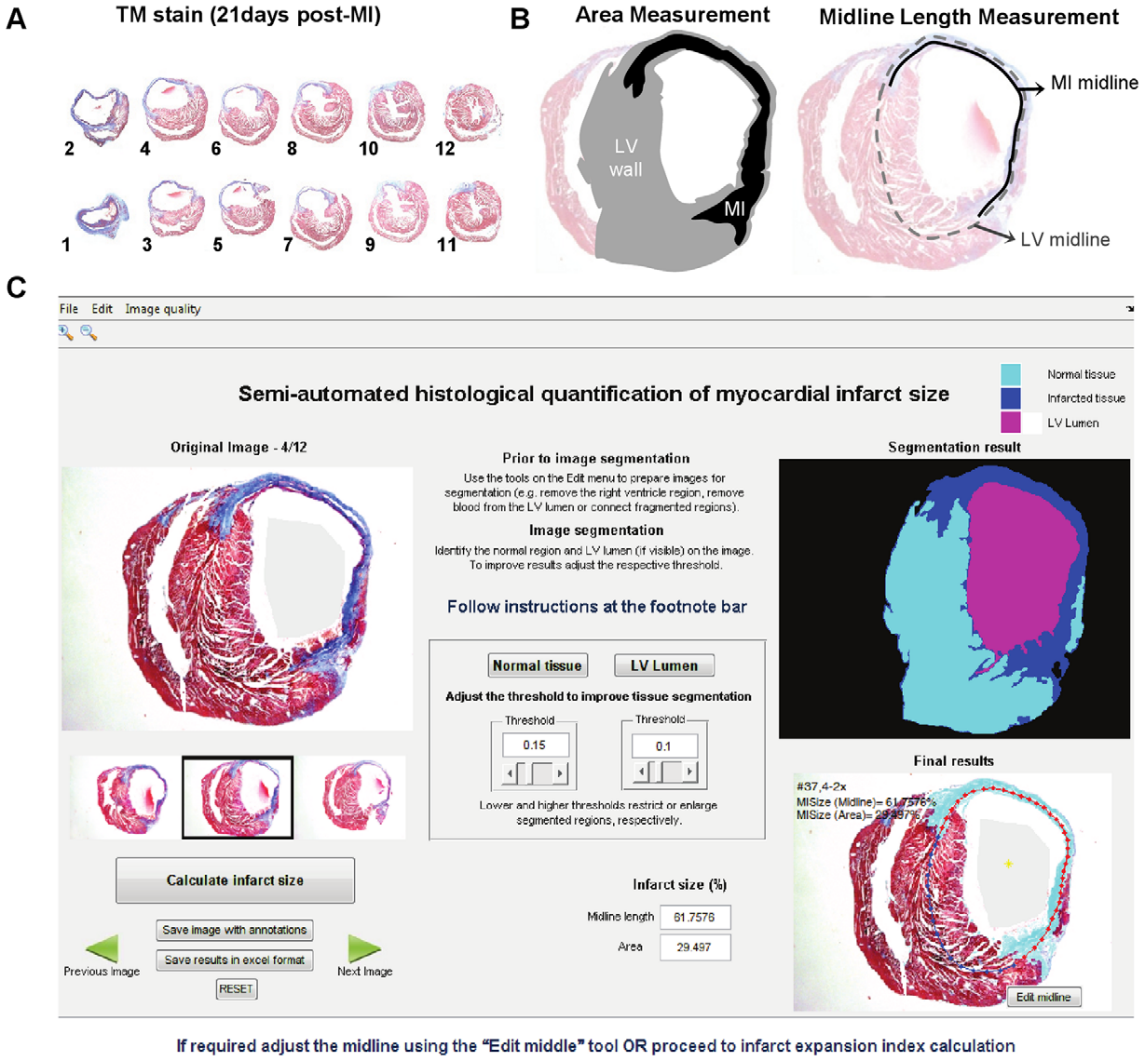
Manual and *MIQuant* semi-automated calculation of MI size in chronic infarcts. (A) LV representative MT stained sections, numbered from the apex to the LV base, were obtained from an infarcted heart harvested at 21 days post-surgery. (B) Histological infarct size calculation by the area method requires manual tracing of the LV myocardium (light gray) and of the scarred LV tissue (black). The infarct size, expressed as a percentage, is the division of the infarct area by the LV area multiplied by 100. For the midline length approach (right) the midline, herein defined as the mid-region between the epicardial and endocardial surfaces, of the total LV (dashed line) and of scarred region (full line) are manually traced. The infarct size, expressed as a percentage, is the division of the infarct midline length by the LV midline length multiplied by 100. The total LV infarct extent is the average of infarct size obtained for the LV representative cross-sections (A). (C) Screen shot of *MIQuant* layout following infarct size calculation. Multiple images can be uploaded in TIFF or JPEG file-formats and the software calculates the intermediate values of infarct size for each image (bottom right). A total MI size is also generated assuming that the uploaded images were representative sections of the LV. For selection of the scarred myocardium (top right) the software requires the user to double-click in a normal tissue region and in the LV lumen, if applicable, over the uploaded image (top left).

Representative sampling of the LV (approx. 12 sections) was obtained by transverse sectioning (3 µm) from the apex to the base (atrium region) of paraffin-embedded hearts with an interval of 300 µm among each section (Figure 1A).

Paraffin sections were stained with modified Masson's trichrome staining (MT). MT staining was performed according to the Trichrome (Masson) Stain kit (Sigma-Aldrich) with the following modifications: nuclei were pre-stained with Celestine Blue solution following staining with Gill's Hematoxylin and incubation for 1 hour in aqueous Bouin's solution to promote a uniform staining.

### Myocardial infarct size calculation

For infarct size determination the collagen deposition, highlighted (blue) in MT-stained sections collected at 21 days post-infarction, was used to define the LV scarred region. Images of histological sections were captured with an Olympus SZX10 stereomicroscope and Olympus DP21 camera. The percentage of affected LV wall was calculated by two different and previously validated methods: the *area measurement* (calculated by dividing the infarct area by the total LV area) [13] and the *midline length measurement* (calculated by dividing the midline length of the infarcted LV wall by the midline length of total LV wall). Only regions with infarct in >50% of the whole thickness of the myocardium were considered for infarct midline [10]. The MI size determination was performed either manually, by drawing points to outline different anatomical/pathological regions using the Image J 1.42 software (Figure 1B), or by using *MIQuant* (Figure 1C).

### Software design

The *MIQuant* software was implemented in MATLAB™ and a MS Windows™ 32-bit compiled version is available online at http://paginas.fe.up.pt/~quelhas/MIQuant/MIQuant.zip. With the objective of developing an approach for automatic infarct size estimation several image processing methodologies were tested [15] and, within all tested semi-supervised methods, region growing was found to work best and also faster, being selected for the final software implementation.

### Data and statistical analysis

To validate *MIQuant*, four expert researchers analyzed five hearts (twelve sections *per* heart) using midline and area methods, manually and with *MIQuant*. All *experts* repeated measures at three distanced moments (one month between 1^st^ and 2^nd^ measure and one week between 2^nd^ and 3^rd^). A one-way repeated measures analysis of variance (ANOVA) was conducted to evaluate repeatability. Seven non-trained *volunteers* measured the same samples using *MIQuant*. The association between manual and *MIQuant* results was investigated using the Pearson product-moment-correlation coefficient (*r*). Additionally, to address agreement amongst methods, the Bland-Altman agreement statistical method was used [16] following verification of the normal distribution (Gaussian) of results. A two-way between-groups ANOVA was applied to address the impact of observers and heart samples in the results. Post-hoc comparison using the Tukey HSD test was performed. Expert and volunteer results were compared by an independent-samples *t*-test. The time required for manual and *MIQuant-*assisted infarct size calculation was compared by the Mann-Whitney test.

## Results

### Software overview and availability

*MIQuant* is a user-friendly software application that assists on the infarct size quantification in an experimental MI-setting. The infarct size, defined as the percentage of the LV affected by coronary artery occlusion, is estimated with representative cross-sections of the LV stained with MT that enables the identification of collagen deposition, a hallmark of established infarction. The software allows the upload of single or multiple images and enables the computation of the MI size of each image, calculated by area [13] and midline length [10] methods, and the total infarct size mean value that can be saved in excel file-format.

*MIQuant* was designed by applying the region growing image segmentation method, which exploits the spatial context of pixels with similar pixel-color properties. The main criterion for the algorithm of region growing is homogeneity, similar pixels (or regions) that are neighbors are joined together. For each image, region growing requires initial image points (or seeds) that define the region of interest. From these initial points the algorithm grows until no more neighbors can be joined to the region of interest, therefore regions/pixels are merged if they satisfy the chosen criterion and no merging occurs when the criterion is not met [17], [18]. In the *MIQuant* software the user is asked to provide input seed points for the LV lumen (if present in the image) and the viable myocardium (if present in the image), prior to automated segmentation. The choice of not requiring the user to select the infarcted LV region has to do with the heterogeneity of the ischemic tissue. The user clicks with the mouse on the heart section image and gives as many input points as desired. Following selection of the viable myocardium and/or LV lumen the segmentation is generated and displayed on the screen. This will be the support for the infarct size computation. User adjustments to the segmentation are accessible by varying the merging criteria and the segmentation process can be repeated until the user is satisfied with the results. When the segmentation is complete the user can request computation of infarct size results by both midline length and area methods. For the midline length measurement, the *MIQuant* software automatically traces lines from the lumen centre outwards and identifies the middle distance between tissue boundaries. The midline of the infarcted region was considered when the LV wall was affected in more than 50% in radial direction. The midline generated by the software can be adjusted by the user prior to MI size calculation.

Commands for image edition are available on the “edit menu”, which permits the removal of tissue regions/artifacts that may interfere with tissue automated segmentation, e.g. the right ventricle or blood within the LV lumen.

The *MIQuant* software was implemented in MATLAB™ and a MS Windows™ 32 bit compiled version is available online at http://paginas.fe.up.pt/~quelhas/MIQuant/MIQuant.zip. The archive should be downloaded and unzipped into a specific folder. The *MIQuant* manual reading is recommended prior to beginning with the software, available at http://www.fe.up.pt/~quelhas/MIQuant/MIQuant_manual.pdf. *MIQuant* requires the installation of MATLAB™ or of the MATLAB™ Component Runtime (MCR) installer (http://paginas.fe.up.pt/~quelhas/MIQuant/MCRInstaller.zip). The application can be initiated by double-click on the executable “MIQuant” file. More information about the software usage and installation is available at the *MIQuant* website http://www.fe.up.pt/~quelhas/MIQuant/.

### MIQuant repeatability and reproducibility

**Figure 2:**
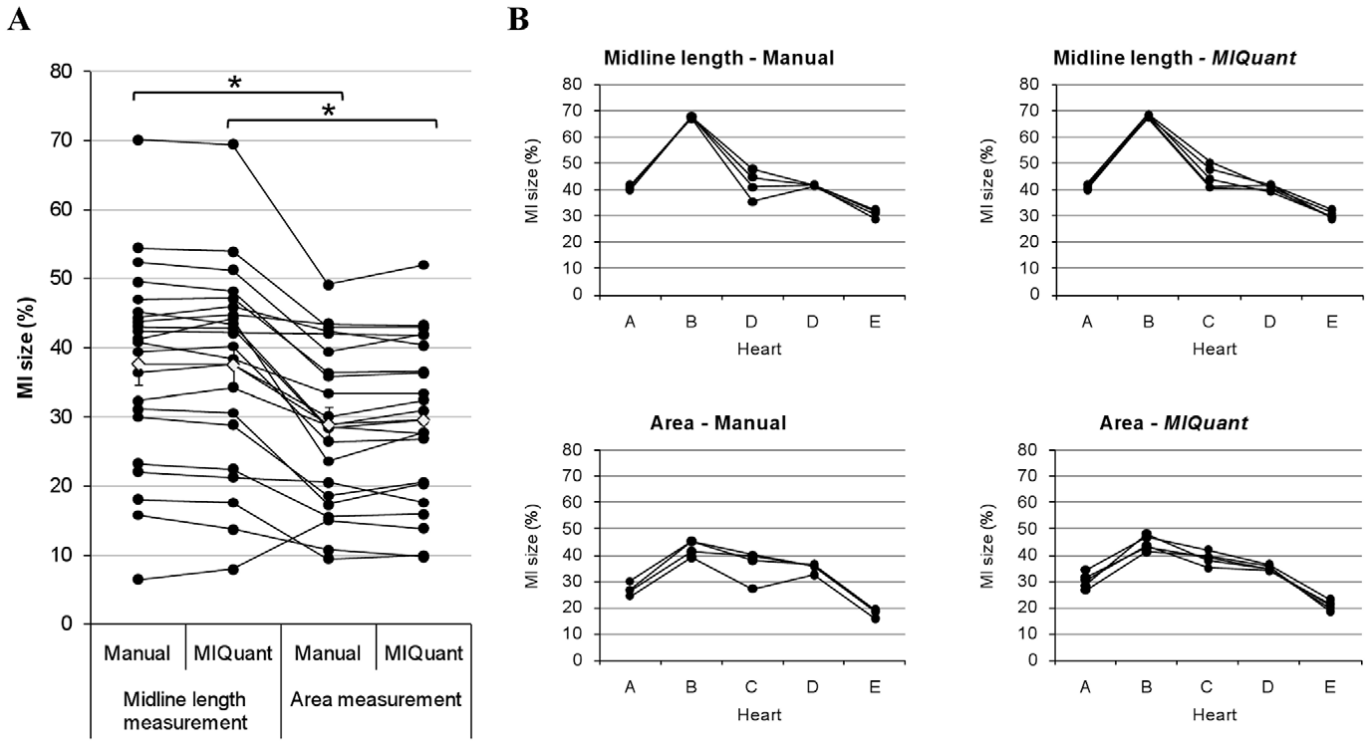
Consistency and reproducibility of *MIQuant infarct size calculation*. (A) Consistency of manual and *MIQuant* infarct size results obtained using the area and midline length measurements. Hearts were harvested at 21 days post-surgery and infarct size determinations are the mean value of 12 cross-sections representative of the LV. Mann-Whitney statistical analysis demonstrated significant differences between the area and midline length methods, as already described by Takagawa [10]. (B) Reproducibility of *MIQuant* measurements. Although ANOVA demonstrated no significant influence of the observer on the LV infarct size scores obtained, neither manually nor using *MIQuant*, the latter displays a tendency for lower discrepancy between operators. ⋄ indicates the mean value of each group. * *p*<0.05.

Manual and *MIQuant* infarct size quantification was assessed by two well-validated methods, i.e. the area and the midline length measurement (Figure 2A). Visual inspection of the infarct size scores across methods demonstrate that *MIQuant* results are consistent with the manual assessment, and thus infarct values obtained with the area measurement were significantly smaller than the midline length infarct scores. The similarity between the manual and *MIQuant* approaches demonstrate that the latter might constitute an alternative for the histological quantification of infarct size. Further validation of *MIQuant* is detailed bellow.

Intra- (repeatability) and inter-observer (reproducibility) variability was considered in the experimental design, thus three independent measures were conducted by four different users. Repeated measures one-way ANOVA was applied to compare the repeatability of the area and midline length-based methods, both calculated manually and by using *MIQuant*. The LV infarct size means with standard deviations and ANOVA results are detailed in table 1. No significant effect of the repetition was found on the infarct size obtained *per* section and *per* heart, i.e. mean value of 12 sections representative of the LV, demonstrating the consistency of *MIQuant* measurements obtained at different instances.

**Table 1:** Repeatability analysis of the manual and *MIQuant* results by repeated measures one-way ANOVA.

MI size (%)	Midline length measurement^1^			Area measurement^2^		
	Measurement 1	Measurement 2	Measurement 3	Measurement 1	Measurement 2	Measurement 3
Manual**^a^**	44.24±13.01	44.84±12.62	45.03±12.82	31.23±9.60	32.51±9.29	31,92±9.25
*MIQuant* ***^b^***	44.66±13.26	44.58±13.00	45.61±13.01	34.02±8.71	34.00±8.16	35,04±8.72

Values area mean ± STDEV; *n* = 20; ^1, a^ ***Per*** **LV**: Wilks'Lambda = 0.818, F(2, 18) = 2.0001, p = 0.164, multivariate partial eta squared = 0.18; ***Per*** **section**: Wilks'Lambda = 0.990, F(2, 234) = 2.0001, p = 0.140, multivariate partial eta squared = 0.322; ^1, b^ ***Per*** **LV**: Wilks'Lambda = 0.757, F(2, 18) = 2.892, p = 0.081, multivariate partial eta squared = 0.24; ***Per*** **section**: Wilks'Lambda = 0.977, F(2, 234) = 2.734, p = 0.067, multivariate partial eta squared = 0.023; ^2, a^ ***Per*** **LV**: Wilks'Lambda = 0.848, F(2, 18) = 1.617, p = 0.226, multivariate partial eta squared = 0.15; ***Per*** **section**: Wilks'Lambda = 0.969, F(2, 234) = 3.737, p = 0.025, multivariate partial eta squared = 0.031; ^2, b^ ***Per*** **LV**: Wilks'Lambda = 0.827, F(2, 18) = 1.886, p = 0.180, multivariate partial eta squared = 0.17; ***Per*** **section**: Wilks'Lambda = 0.981, F(2, 234) = 2.286, p = 0.104, multivariate partial eta squared = 0.019.

Inter-observer variability for each analyzed sample is displayed on Fig. 2B. A two-way ANOVA was conducted to investigate whether the observer influences (inter-observer variability) infarct size measurements manually or using *MIQuant*. Post-hoc comparison using the Tukey HSD test indicated that the mean score of infarct size, for each heart, did not differ significantly (*p*>0.05) among observers in any of the tested infarct size quantification methods. However, a tendency for increased variability of the manual results when compared to *MIQuant* was observed and was particularly evident on heart C, which is the sample that retrieved more deviation amongst users (Figure 2B).

### Validation of MIQuant infarct size quantification

**Figure 3:**
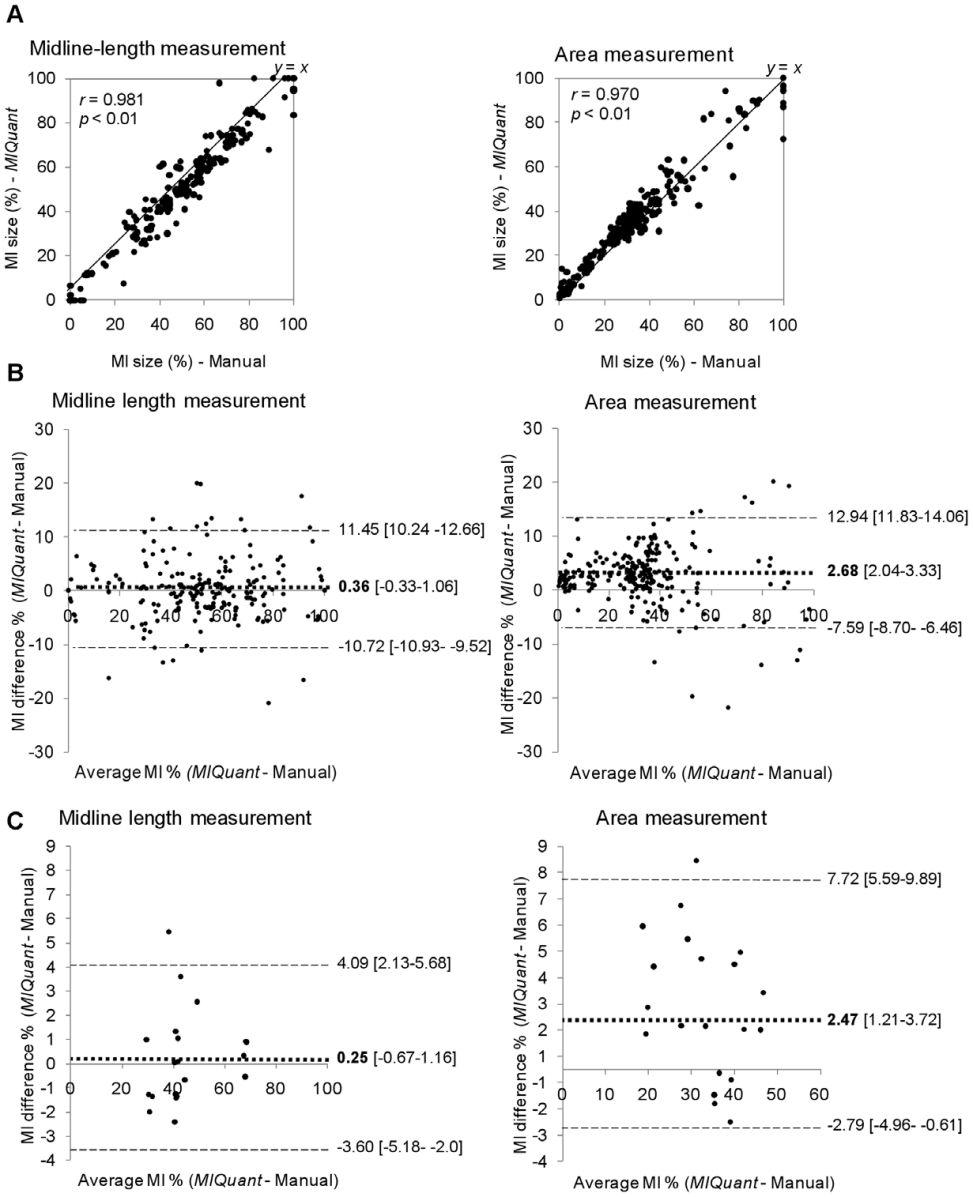
Validation of *MIQuant* for infarct size assessment. (A) Infarct size scatter diagram and the Pearson coefficient demonstrated strong association between manual and *MIQuant* results for area (*right*) and midline length (*left*) approaches. (B) Bland-Altman concordance analysis of the manual and *MIQuant* infarct size measurements demonstrated acceptable limits of agreement between methods. Average values of the three independent measures of infarct size *per* section (B) and *per* heart (C) were subject to the analysis. Differences between the infarct sizes retrieved by each method (*MIQuant*-manual) are displayed in the *y*-axis and the mean infarct size values are plotted in the *x*-axis. The limits of agreement (- -) and bias (▪▪▪) and respective 95% confidence intervals ([ ]) are shown.

A scatter diagram of the infarct size values measured manually and by *MIQuant* is shown in Fig. 3A. The Pearson Product-moment correlation for the individual data points was *r* = 0.981 for the midline length and *r* = 0.970 for the area methods, with a significance level of *p*<0.01, hence the infarct size values obtained by *MIQuant* are strongly associated to the manual quantification. The strong correlation between manual and *MIQuant* results prompted further analysis to evaluate the magnitude and direction of the differences between methods.

The gold-standard statistical analysis applied to method-comparison studies is the Bland-Altman plot, which determines the agreement of two methods that measure the same variable [16], [19]. Manual and *MIQuant* results were subjected to the Bland-Altman agreement statistical method that predicts the bias, i.e. difference in values obtained by the two methods, and the limits of agreement between methods (Figure 3B and C). Bias and concordance limits of ±2% and ±7%, respectively, were established *a priori* as the maximum parameters for acceptance of *MIQuant* regarding *per* heart infarct size quantifications. These values were selected on the basis of acceptance limits addressed for infarct size methods on published studies [20], [21], [22], [23]. The *a priori* establishment of acceptable agreement limits for infarct size *per* section was conditioned by the fact that, to our best knowledge, no previous comparison was performed for single sections. Hence, since it is expected higher degree of discordance across sections, when compared to the mean value, a low-stringency predetermined bias and concordance limits of ±2% and ±15%, respectively, were established.

Bland-Altman analysis was conducted with manual and *MIQuant* results obtained *per* LV section (Figure 3B). The estimated bias is 0.36% with concordance limits of −10.72% and 11.45% for the midline length method, whereas for the area approach the bias is 2.68% with limits of agreement of −7.59% and 12.94% (Figure 3B). Hence, for both methodological approaches, the predicted confidence interval is within acceptance limits and so *MIQuant* is considered equivalent to the established manual quantification method.

The visual inspection of Bland-Altman plot denoted that differences between *MIQuant* and manual measurements are scattered around the bias with no obvious pattern for the midline length results whereas, the area differences appear to increase for higher infarction values (Figure 3B). To determine whether an association exists between the methods discrepancies and the size of infarction, the Pearson coefficient was calculated and a small, non-statistically significant correlation between the two variables was observed (*r* = 0.063; *p* = 0.337).

Measurements of the infarct size *per* heart, i.e. mean value of 12 sections representative of the LV, obtained by the manual and *MIQuant* calculation were also compared accordingly to the Bland-Altman concordance analysis. For the midline length the predicted bias is 0.25% and the limits of agreement are −3.60% and 4.09%, resulting on 7.74% amplitude of concordance (Figure 3C). The analysis of the area measurements retrieves a mean difference of 2.47% (95% confidence interval (CI) from 1.21% to 3.72%), suggesting that *MIQuant* tends to give a higher reading from 1.21% to 3.72% (Figure 3C). The area method concordance interval ranges from −2.79% to 7.72%. Thus, for *MIQuant per* heart infarct size results the confidence interval of the predicted bias and concordance limits are within acceptance limits (bias ±2%, concordance limits ±7%) for both midline length- and area-measurements, which show that the performance of *MIQuant* is equivalent to the manual infarct size calculation.

Although the differences between *MIQuant* and manual measurements are scattered around the bias with no obvious pattern, the association between the two variables was investigated using the Pearson Product-moment-correlation coefficient. Small and non-statistically significant correlations were found for both midline length (*r* = 0.149; *p* = 0.531) and area (*r* = −0.315; *p* = 0.176) approaches, consequently discrepancies between the manual and semi-automated quantification are independent of the sample infarction size.

### Validation of MIQuant by non-trained volunteer-users

**Figure 4:**
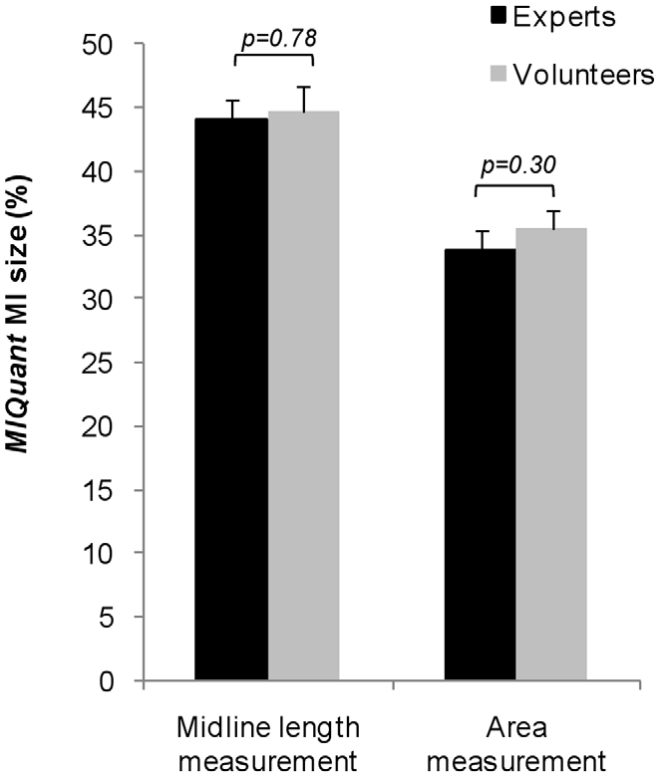
*MIQuant* efficacy is not affected by user proficiency. *MIQuant* infarct size values obtained by competent (*experts*) and non-trained (*volunteer*) users were compared and the mean values are displayed as graph bars. Independent-samples *t*-test showed no significant differences between infarct scores calculated by the *experts vs. volunteers*; furthermore, a two–way ANOVA demonstrated no significant influence of the user on the obtained infarct size value.

To address whether previous experience with the *MIQuant* application and knowledge on infarct size calculation are strict requirements for the correct software usage, a comparison was established between *MIQuant* results obtained by users with distinct proficiency. Five hearts were independently analyzed by four competent users (*experts*), i.e. investigators with extensive training on MI size quantification either manually or using *MIQuant*, and by volunteer-users with no previous experience on MI size calculation, but to whom free-access to the *MIQuant* manual was provided. An independent-samples *t*-test was conducted and no significant differences were observed on the midline length and area measurements obtained by either *experts* or *volunteers* (Figure 4). In addition, a two–way ANOVA analysis of variance was conducted to explore the impact of the observer type (expert or volunteer) and the heart sample on *MIQuant* infarct size measurement, obtained by either midline length (ML) or area (A) approaches. There was no statistically significant effect for the observer type (ML *p* = 0.267; A *p* = 0.77), whereas the effect of the heart sample was found to be statistically significant (*p*<0.05).

### Time-efficiency of MIQuant infarct size quantification

**Figure 5:**
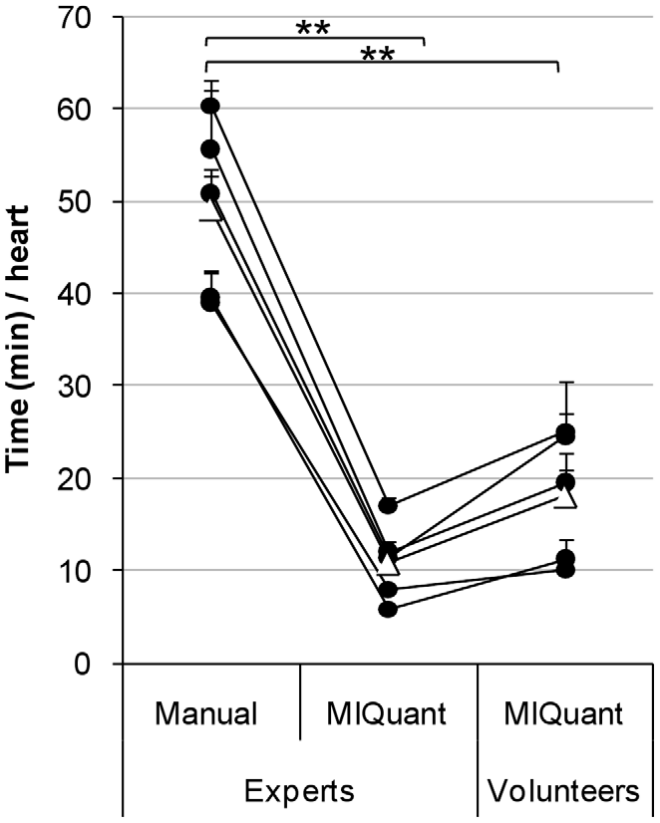
*MIQuant* improves the time-efficiency of infarct size quantification. The time consumption of the infarct size determination *per* heart (mean value of 12 representative sections of the LV) was compared between the manual and *MIQuant* approaches. The Δ indicates the mean value of each group. **p<0.01.

The manual quantification of MI size is a time-consuming and laborious endeavor, thus the simplification of this task is highly desired and was a major drive for the development of *MIQuant*. The time required for manual and *MIQuant-*assisted infarct size calculation was compared (Figure 5). The latter was additionally compared for *experts* and volunteer operators. Despite the required definition of initial parameters by the user prior to *MIQuant* segmentation, this method resulted on a significant overall 4.5- and 3-fold decrease in the time period spent on the analysis when performed by competent and volunteer users, respectively.

## Discussion

In this study, the development and validation of *MIQuant*, a simple and user-friendly software application that calculates the infarct size on cardiac models of induced-ischemia, is reported. To our best knowledge, *MIQuant* constitutes the first computer-assisted tool to ease the arduous and time-consuming endeavor of manual infarct size calculation by classical planimetry.

The view of the heart as a post-mitotic organ has been challenged in recent years by reports of cardiomyocyte renewal in humans [24], cardiomyocytic-cell replacement after injury in mouse [25] and of myocardium-resident Sca-1^+^/c-Kit^+^/MDR1^+^ progenitor/stem-like cells [26], [27], [28]. These findings, together with the fact that cardiovascular diseases are a major cause of morbidity/mortality, have encouraged the publication of studies on the evaluation of cardio-regenerative potential of novel therapies. The latter are commonly tested on rodent models of MI and the infarct size has been regarded as a decisive parameter for the determination of the success of the therapy under test. Although histological planimetry is the gold standard for infarct size quantification, methodological discrepancies are frequent across publications due to a general lack of standardized protocols/methods. The most common methods used to quantify infarct extension are either based on the infarcted area or on the length of the infarction circumference. Both methodologies show limitations related to the infarct size estimation accuracy using parameters that are affected and distorted by cardiac remodeling subsequent to MI [29]. Regarding *MIQuant*, we decided to make available two methods for infarct quantification: the area-based quantification first described by Michael [13] and the midline length measurement that was extensively validated recently [10]. In accordance with Takagawa's [10] observations on manual infarct size quantification, with *MIQuant* we obtained a statistically significant compression of the area results when compared to the midline-length method. Overall, obvious consistency was achieved between manual and *MIQuant* infarct size quantification, which was further illustrated by the excellent correlation between both and by Bland-Altman analysis. Bland-Altman analysis indicated good agreement free from systematic bias for midline-length *MIQuant* infarct scores (0.25±3.84). Regarding the area measurements, although *MIQuant* overestimates infarct size by 1.21–3.27% as compared to the manual quantification, the biological relevance of this overestimation is negligible. Moreover, a random dispersion of results around the predicted bias was observed, demonstrating that *MIQuant* results are reliable independently of the size of infarction. The repeatability and reproducibility of *MIQuant* results were also confirmed by the use of three independent measures obtained by four independent observers. Overall these results indicate that *MIQuant* is a reliable alternative to the manual quantification of infarct size.

Despite being a determinant factor for an accurate estimation of the infarct size[10], the number of transverse sections used for such analysis is extremely variable across studies. One of the advantages of *MIQuant* over the classical manual quantification is the 4.5 fold reduction on the time spent on the analysis, thus improving time-efficiency and allowing the investigator to increase the number of sections *per* analysis and consequently the accuracy of results.

*MIQuant* is available as freeware for research use. The widespread use of *MIQuant* will constitute by itself a major improvement towards normalization of infarct size assessment by restricting the methods to the area and midline length, by standardizing the histological stain used and by restricting the criteria for the identification of the infarcted region. Our results also indicated a tendency, although not statistically significant, for reduced inter-observer variability in *MIQuant* infarct size scores when compared to manual analysis. This may well be underestimated given that the observers in this study were investigators that received similar training on infarct size calculation. It is therefore expected that the diversity of criteria on infarct identification/calculation of observers with different backgrounds will result in increased variability for the manual outcome. In contrast, we demonstrated that *MIQuant* efficacy is independent of previous training with the software and experience on MI size calculation. An interesting experiment would be a comparative analysis between *MIQuant* and manual quantification with experts from different laboratories to therefore undoubtedly clarify whether *MIQuant* contributes to the homogenization of infarct size results. Our attempts to engage in this task *experts* with previous published work on infarct size histological quantification, met with little success and the intent was therefore aborted.

For the interpretation of this study several limitations should be considered: firstly a single species (mouse) was used for the validation of *MIQuant*, and secondly the only model of cardiac induced-ischemia performed was the permanent LAD coronary artery ligation. However, the pathophysiological and morphological alterations following MI are similar in the rat and the mouse [9], [30], [31], supporting the applicability of *MIQuant* for the quantification of rat infarcts. The extension of *MIQuant* to other infarction models, e.g. ischemia-reperfusion or the cryoinjury, is of major interest. Hence, because the software recognizes the infarction region by the collagen deposition, a hallmark of established infarction, we are confident on the software applicability to other models. Indeed, in hearts with non-transmural infarction that very much resembles the reperfusion scenario, *MIQuant* infarct scores were similar to manual quantification (data not shown).

We conclude that *MIQuant* is a valid and easy-to-use software application that assists on infarct size calculation. The widespread use of *MIQuant* will contribute to the reduction of time spent on the analysis and for the standardization of infarct size quantification across studies and, therefore, to a more systematic evaluation of the cardiac regenerative potential of newly developed therapies.

## References

[1] Lloyd-Jones D, Adams RJ, Brown TM, Carnethon M, Dai S (2010). Executive summary: heart disease and stroke statistics–2010 update: a report from the American Heart Association.. Circulation.

[2] Gonzales C, Pedrazzini T (2009). Progenitor cell therapy for heart disease.. Experimental Cell Research.

[3] Zaragoza C, Gomez-Guerrero C, Martin-Ventura JL, Blanco-Colio L, Lavin B (2011). Animal models of cardiovascular diseases.. J Biomed Biotechnol.

[4] Sun Y (2009). Myocardial repair/remodelling following infarction: roles of local factors.. Cardiovasc Res.

[5] Ramani R, Nilles K, Gibson G, Burkhead B, Mathier M (2011). Tissue Inhibitor of Metalloproteinase-2 Gene Delivery Ameliorates Postinfarction Cardiac Remodeling.. Clinical and Translational Science.

[6] Ahmed RP, Haider KH, Shujia J, Afzal MR, Ashraf M (2010). Sonic Hedgehog gene delivery to the rodent heart promotes angiogenesis via iNOS/netrin-1/PKC pathway.. PLoS One.

[7] Smits AM, van Laake LW, den Ouden K, Schreurs C, Szuhai K (2009). Human cardiomyocyte progenitor cell transplantation preserves long-term function of the infarcted mouse myocardium.. Cardiovasc Res.

[8] Yoon YS, Wecker A, Heyd L, Park JS, Tkebuchava T (2005). Clonally expanded novel multipotent stem cells from human bone marrow regenerate myocardium after myocardial infarction.. J Clin Invest.

[9] Pfeffer MA, Pfeffer JM, Fishbein MC, Fletcher PJ, Spadaro J (1979). Myocardial infarct size and ventricular function in rats.. Circ Res.

[10] Takagawa J, Zhang Y, Wong ML, Sievers RE, Kapasi NK (2007). Myocardial infarct size measurement in the mouse chronic infarction model: comparison of area- and length-based approaches.. J Appl Physiol.

[11] Gao X-M, Dart AM, Dewar E, Jennings G, Du X-J (2000). Serial echocardiographic assessment of left ventricular dimensions and function after myocardial infarction in mice.. Cardiovascular Research.

[12] Patten RD, Aronovitz MJ, Deras-Mejia L, Pandian NG, Hanak GG (1998). Ventricular remodeling in a mouse model of myocardial infarction.. American Journal of Physiology - Heart and Circulatory Physiology.

[13] Michael LH, Entman ML, Hartley CJ, Youker KA, Zhu J (1995). Myocardial ischemia and reperfusion: a murine model.. Am J Physiol.

[14] Minicucci MF, Azevedo PS, Duarte DR, Matsubara BB, Matsubara LS (2007). Comparison of different methods to measure experimental chronic infarction size in the rat model.. Arq Bras Cardiol.

[15] Esteves T, Valente M, Nascimento DS, Perpétua PO, Quelhas P (2011). Automatic and semi-automatic analysis of the extension of myocardial infarction in an experimental murine model..

[16] Bland JM, Altman DG (1986). Statistical methods for assessing agreement between two methods of clinical measurement.. Lancet.

[17] Wu Q, Merchant F, Castleman K, 7 C (1996). Microscope Image Processing;.

[18] Gonzalez R, Woods R, Eddins S (2004). Digital Image Processing Using MAT-LAB.

[19] Hanneman SK (2008). Design, analysis, and interpretation of method-comparison studies.. AAN Adv Crit Care.

[20] Bohl S, Lygate CA, Barnes H, Medway D, Stork L-A (2009). Advanced methods for quantification of infarct size in mice using three-dimensional high-field late gadolinium enhancement MRI.. American Journal of Physiology - Heart and Circulatory Physiology.

[21] Yang Z, Berr SS, Gilson WD, Toufektsian M-C, French BA (2004). Simultaneous Evaluation of Infarct Size and Cardiac Function in Intact Mice by Contrast-Enhanced Cardiac Magnetic Resonance Imaging Reveals Contractile Dysfunction in Noninfarcted Regions Early After Myocardial Infarction.. Circulation.

[22] Dawson D, Lygate CA, Saunders J, Schneider JE, Ye X (2004). Quantitative 3-Dimensional Echocardiography for Accurate and Rapid Cardiac Phenotype Characterization in Mice.. Circulation.

[23] Protti A, Sirker A, Shah AM, Botnar R (2010). Late gadolinium enhancement of acute myocardial infarction in mice at 7T: Cine-FLASH versus inversion recovery.. Journal of Magnetic Resonance Imaging.

[24] Bergmann O, Bhardwaj RD, Bernard S, Zdunek S, Barnabe-Heider F (2009). Evidence for cardiomyocyte renewal in humans.. Science.

[25] Hsieh PC, Segers VF, Davis ME, MacGillivray C, Gannon J (2007). Evidence from a genetic fate-mapping study that stem cells refresh adult mammalian cardiomyocytes after injury.. Nat Med.

[26] Barile L, Messina E, Giacomello A, Marban E (2007). Endogenous cardiac stem cells.. Prog Cardiovasc Dis.

[27] Beltrami AP, Barlucchi L, Torella D, Baker M, Limana F (2003). Adult cardiac stem cells are multipotent and support myocardial regeneration.. Cell.

[28] Leri A, Kajstura J, Anversa P (2005). Cardiac Stem Cells and Mechanisms of Myocardial Regeneration.. Physiol Rev.

[29] Zornoff LA, Paiva SA, Minicucci MF, Spadaro J (2009). Experimental myocardium infarction in rats: analysis of the model.. Arq Bras Cardiol.

[30] Fishbein MC, Maclean D, Maroko PR (1978). Experimental myocardial infarction in the rat: qualitative and quantitative changes during pathologic evolution.. Am J Pathol.

[31] Virag JI, Murry CE (2003). Myofibroblast and Endothelial Cell Proliferation during Murine Myocardial Infarct Repair.. Am J Pathol.

